# The Potential Advantage of Targeting Both PD-L1/PD-L2/PD-1 and IL-10–IL-10R Pathways in Acute Myeloid Leukemia

**DOI:** 10.3390/ph14111105

**Published:** 2021-10-29

**Authors:** Laura Jimbu, Oana Mesaros, Alexandra Neaga, Ana Maria Nanut, Ciprian Tomuleasa, Delia Dima, Corina Bocsan, Mihnea Zdrenghea

**Affiliations:** 1 Department of Hematology, Iuliu Hatieganu University of Medicine and Pharmacy, 8 Babes Str., 400012 Cluj-Napoca, Romania; mesaros.oana@umfcluj.ro (O.M.); Neaga.alexandra@umfcluj.ro (A.N.); ana.maria.nanut@elearn.umfcluj.ro (A.M.N.); ciprian.tomuleasa@umfcluj.ro (C.T.); mzdrenghea@umfcluj.ro (M.Z.); 2 Department of Hematology, Ion Chiricuta Oncology Institute, 34-36 Republicii Str., 400015 Cluj-Napoca, Romania; deli_dima@yahoo.com; 3 “Octavian Fodor” Regional Institute of Gastroenterology and Hepatology, 19-21 Croitorilor Str., 400162 Cluj-Napoca, Romania; 4 Department of Clinical Pharmacology, Iuliu Hatieganu University of Medicine and Pharmacy, 8 Babes Str., 400012 Cluj-Napoca, Romania; bocsan.corina@umfcluj.ro

**Keywords:** PD-1, PD-L1, IL-10, acute myeloid leukemia, cancer

## Abstract

Tumor cells promote the suppression of host anti-tumor type 1 T cell responses by various mechanisms, including the upregulation of surface inhibitory molecules such as programmed death ligand (PD-L)-1, and the production of immunosuppressive cytokines such as interleukin-10 (IL-10). There are over 2000 trials investigating PD-L1 and/or its receptor programmed-death 1 (PD-1) blockade in cancer, leading to the approval of PD-1 or PD-L1 inhibitors in several types of solid cancers and in hematological malignancies. The available data suggest that the molecule PD-L1 on antigen-presenting cells suppresses type 1 T cell immune responses such as cytotoxicity, and that the cytokine IL-10, in addition to downregulating immune responses, increases the expression of inhibitory molecule PD-L1. We hypothesize that the manipulation of both the co-inhibitory network (with anti-PD-L1 blocking antibodies) and suppressor network (with anti-IL-10 blocking antibodies) is an attractive immunotherapeutic intervention for acute myeloid leukemia (AML) patients ineligible for standard treatment with chemotherapy and hematopoietic stem cell transplantation, and with less severe adverse reactions. The proposed combination of these two immunotherapies represents a new approach that can be readily translated into the clinic to improve the therapeutic efficacy of AML disease treatment.

## 1. Introduction

Mechanisms exploited by tumor cells to inhibit CD8 T-cell-mediated immunity include the disruption of antigen presentation, downregulation of human leukocyte antigenmolecules, and induction of co-inhibitory molecules such as programmed death ligand (PD-L)-1 (B7 homolog 1 (B7-H1); CD274) and -2 (PD-L2; B7-DC; CD273). PD-L1 and PD-L2 are both members of the B7 family.

PD-L1 molecules are constitutively expressed by a range of hematopoietic cells such as dendritic cells (DC), monocytes/macrophages, and non-hematopoietic cells (such as fibroblasts, endothelial cells, and epithelial cells) [1,2,3] and they are increased by pro-inflammatory stimuli such as lipopolysaccharides (LPS), type I interferons (IFNs), type 1 cytokine IFN-γ, polyinosinic:polycytidylic acid, and viruses [4,5,6,7,8,9]. We reported that blocking PD-L1 antibodies in a co-culture system of virus-infected epithelial cells with purified CD8 T cells enhanced CD8 T cell type 1 immune responses (IFN-γ, IL-2, and granzyme B production) and decreased virus load [10].

PD-L2 molecule levels are low in DC, macrophages, activated T cells, endothelial cells, and epithelial cells and are increased by IL-4, Toll-like receptor (TLR) ligands, and viruses [11,12,13,14,15,16,17,18,19].

During inflammation, both PD-L1 and PD-L2 ligands are increased by cytokines present in the milieu, PD-L2 later and at lower levels. They cross-compete for the same co-inhibitory receptor; PD-1 (CD279) on antigen-specific activated T cells, and PD-L2 with higher affinity than PD-L1 [3,11,20,21,22,23]. PD-1, a distant member of the CD28 family, is an immunoreceptor with a tyrosine-based switch motif and an inhibitory motif in its cytoplasmic tail, upregulated in response to T cell receptor triggering, and signaling inhibition for proliferation, IL-2 and IFN-γ cytokine production, cytolytic function, and survival of the T cell, increasing IL-10 production [11,24,25,26,27]. However, PD-1, by limiting STAT-1 phosphorylation, is involved in the negative regulation of IL-12 production by PD-L-positive human monocytes/macrophages [28,29] and cells are rendered resistant to T-cell-mediated and FasL-mediated lysis by PD-1 signaling cell-expressed PD-L1 [30]. The role of PD-1 in signaling without association with an antigen receptor is not clear.

PD-L1 could bind to a second receptor, B7-1/CD80, which also transduces inhibitory signals into T cells in vitro and in vivo [31,32]. Because CD80 and PD-1 bind to the same region of PD-L1, it was suggested that PD-1 could compete with CD80 for binding to PD-L1 [31]. Additionally, a homolog of PD-1, named PD-1 homolog (PD-1H), has been discovered [33,34]. PD-1H is broadly expressed on the cell surface of hematopoietic cells and could be further upregulated on T cells following activation. Importantly, PD-1H expression on tumor cells resulted in diminished antitumor immunity.

PD-L2 also binds PD-1 and it has been reported that PD-L2 upregulates T cell proliferation and IFN-γ production independent of the PD-1 receptor [21,35]. In contrast to PD-L1, PD-L2 molecules augment T helper 1 and cytotoxic T lymphocytes (CTL) responses 1 and inhibit type 2 responses, both during the induction and the effector phase, and blocks IL-10 production [36,37,38]. PD-L2 attenuated strong Th2 responses induced by Nippostrongylus brasiliensisas via an unknown alternative T cell receptor that enhances Th1 responses, which is required for effective anticancer immunity, and enhanced disease severity was reported when PD-L2 inhibitors were used, but not when PD-1 blockers were used [15].

These data suggest that relative levels of expression of PD-L1/PD-L2 have roles in regulating tissue type 1/type 2 immune responses in diseases with a pathogenesis involving a type 1/type 2 cytokine production imbalance.

PD-L expression in cancer cells has been shown to inhibit the activity of cytotoxic CD8 T cells. We propose that a combination immunomodulatory therapy blocking the PD-1–PD-L1 pathway coupled with therapy blocking the IL-10–IL-10Receptor (R) pathway will enhance type 1 T cell functions such as cytotoxicity and will shift the immunosuppressive environment in AML towards a type 1 immune-enhancing environment, removing tumor cells.

## 2. IL-10

IL-10 is a 37 kDa protein, produced by several immune cells, such as DC, T regulatory cells (Tregs), macrophages, B, T, and NKT cells, and has the ability to modulate the adaptive and innate immune responses. It is one of the anti-inflammatory cytokines along with IL-4, IL-11, and IL-13 [39] and plays an important role in reducing inflammation and tissue damage in the setting of different types of infections [40]. Its anti-inflammatory role is demonstrated in animal models, in knockout mice that develop inflammatory bowel diseases [41]. IL-10 exerts its function on a plethora of cells. It interferes with DC maturation and inhibits the formation of Th-1 cells, shifting the balance towards a Th2 response [42]; it inhibits the proliferation and activation of macrophages via STAT1 and STAT3 [43]; and it inhibits the activation of CD4^+^ T cells and the production of pro-inflammatory cytokines [44]. The effect of IL-10 on cytotoxic T cells is bidirectional: in association with IL-2 or IL-4, it stimulates the production of CD8^+^ T cells [45], while alone it produces anergy by downregulating the major histocompatibility complex (MHC), class II expression on the antigen-presenting cells (APC), and CD28 [44]. On the other hand, IL-10 increases the cytotoxicity of NK cells and enhances IFN-γ production [46], and stimulates mast cell [47], B cell, and thymocyte proliferation and activation [48]. The timing of IL-10 production is very important. While in most cases, IL-10 protects the host from an exuberant immune response, if produced early during infections it harms the host and promotes fulminant infections [40].

In cancer, the paradigm is different. Several studies showed conflicting results regarding the IL-10 effect on tumors (immunosuppressive vs. immunostimulating activity) [49].

### 2.1. IL-10 and Tumor Progression

Several studies have shown that IL-10 leads to tumor progression due to its ability to downregulate the immune system. A study on gastric tumor tissue showed that IL-10 levels were elevated compared to normal tissue and that it stimulates tumor proliferation and migration and inhibits apoptosis. Additionally, serum IL-10 levels in patients with gastric cancer were higher than in healthy patients [50]. Zhang et al. also showed that in peripheral T cell lymphoma with elevated IL-10 levels (>3.6 pg/mL), complete remission (CR) rates are lower and there is a higher rate of relapse [51]. Additionally, in non-small cell lung carcinoma (NSCLC), it has been shown that a high expression of IL-10 in tumor cells promotes metastasis via angiogenesis and the inhibition of apoptosis [52]. Others showed that a high expression of IL-10 in tumor-associated macrophages (TAMs) is correlated with an adverse prognosis and a more advanced tumor stage, but no significant difference has been seen between IL-10 expression in tumor cells and stage [53].The same results, suggesting a worse prognosis in patients with pancreatic cancer and high levels of IL-10, are presented by Feng et al. in their retrospective study [54]. A meta-analysis comprising 1788 patients with cancer demonstrated that high levels of IL-10, in serum, are correlated with a lower overall survival (OS) both in solid cancers and hematological malignancies [55].

### 2.2. IL-10 as an Anti-Tumor Cytokine

There are several studies that showed that a low or absent expression of IL-10 is associated with poor prognosis. A retrospective study comprising 133 patients with stage I NSCLC showed that patients with restricted IL-10 expression have a worse prognosis than those with retained expression [56]. Another study on colorectal cancer tissue showed that low IL-10 levels in tumor cells are associated with worse prognosis, and with metastasis and invasion [57].

A case report on a patient with refractory lung adenocarcinoma presented a very good response to a PD-L1 inhibitor, nivolumab, associated with pegylated IL-10, at the cost of anemia [58].

While at low concentrations IL-10 is associated with immunosuppression, at higher concentrations IL-10 is correlated with the proliferation and activation of CD8^+^ cells [59]. Additionally, in their mouse model study, Tanikawa et al. demonstrated that IL-10 inhibits tumor development and progression via inhibiting the production of myeloid-derived suppressor cells (MDSCs) and Tregs [60].

Based on these observations, a phase I clinical trial was developed. Patients with different types of solid cancers received pegylated IL-10 in different doses varying from 20 to 40 micrograms/kg. After the administration of pegylated IL-10, proinflammatory cytokines were increased and immunosuppressive cytokines were decreased. The overall response rate (ORR) was 21% [61].

In relapsed pancreatic cancer, a phase III clinical trial showed no statistical difference between FOLFOX (folinic acid + fluorouracil + oxaliplatin) in association with pegylated IL-10 compared to FOLFOX alone [62]. Unfortunately, in metastatic NSCLC patients, the addition of IL-10 to PD-L1 inhibitors, either nivolumab or pembrolizumab, did not bring any benefit [63].

These studies suggest the importance of selecting the “right” patients that could benefit from the addition of pegylated IL-10.

### 2.3. IL-10 and AML

In 1995, Bruserud et al. showed in in vitro studies that the proliferation and the formation of blast colonies is inhibited by IL-10 [64]. Later, in 1998, the same author stated that IL-10, together with IL-13 and IL-4, inhibits the production of cytokines by AML blasts [65]. The same conclusion was drawn in 1996, by Westermann and his colleagues [66]. Additionally, IL-10 demonstrated its ability to inhibit granulocyte colony-stimulating factor (G-CSF) and granulocyte/macrophage colony-stimulating factor (GM-CSF) production by AML blasts, thus inhibiting their autocrine proliferation [67].

In AML, there is a cytokine deregulation due to the abnormal production of cytokines by the blasts and also due to the host’s response to the disease. A study on 42 AML patients measured the levels of several cytokines compared to healthy volunteers. The study showed that IL-4, IL-10, IL-6, IL-5, IL-8, IL12p70, and TNF-α levels were higher in AML patients compared to healthy volunteers, while for IL2, IL-1-β, IL17A, and IFN-γ no significant differences were found. The same study showed that high levels of IL-10 were associated with prolonged OS and event-free survival (EFS) [68]. Kornblau et al. showed that high levels of serum IL-10 in patients with AML were associated with a higher CR, but, interestingly, in this cohort of patients IL-10 levels were lower than in healthy volunteers [69].

On the other hand, some studies suggest that IL-10 may have a tumor-supporting role. A study performed on 46 patients with AML showed that patients with high levels of IL-10 (≥125 pg/mL) had a significantly lower OS (11 months vs. 32 months) [70]. It has been shown that plasmatic IL-10 levels are much higher in newly diagnosed patients with AML compared to patients who achieved CR, suggesting its role in leukemogenesis [71]. Several other studies suggested that IL-10 promotes immunosuppression and thus disease progression [72,73].

A study comprising 131 patients with acute leukemia (of which 95 with AML) assessed intracellular levels of IL-4, IL-10, and IFN-γ in the blast cells and in bone marrow T cells via flow cytometry. There was no significant difference between the level of these cytokines and CR or relapse rate. It was noted, however, that the levels of investigated cytokines normalized once CR was achieved [74].

IL-10R comprises two subunits: IL-10R1 and IL-10R2 [75]. IL-10R2 is expressed on most cells but has low affinity towards IL-10, while IL-10R1 is expressed mostly on hematopoietic cells and in some non-hematopoietic cells such as fibroblasts, epidermal cells, or cytotrophoblasts [76]. IL-10–IL-10R interaction takes place via the JAK/STAT pathway, mostly via IL-10R1, as IL-R2 has a minor role in signal transduction [77]. IL-10R is also expressed on AML blast cells, and when overexpressed it is associated with lower OS. Based on these observations, Chen et al. suggested that IL-10R should be taken into consideration as a target for future immunotherapies [78]. There is not much information regarding the implication of IL-10R in AML, so further investigation would be of high value.

## 3. PD-1–PD-L1 Pathway Suppresses the Type 1 T-Cell Responses Which Fight Cancer

Optimal anti-tumor CD8 T cell responses are predominantly type 1 [79,80,81].

Antigen-specific T cell responses are regulated by co-inhibitory molecules such as PD-L, categorized as “checkpoint molecules” [82]. The expression of PD-L1 molecules on cancer cells is a primary escape mechanism by which tumor cells escape and suppress host immunity, and PD-L1 molecules are being actively investigated as therapeutic targets in solid tumors.

PD-Ls inhibit CD8 T cell activation more effectively than CD4 T cell activation [25] and PD-L1 inhibits CD8 T-cell-mediated cytolysis [83,84,85]. In peripheral tissues, fully activated effector T cells contact the target, such as cancer cells, and PD-L1 on these target cells delivers signals to activate or suppress T cell responses through PD-1. In this way, PD-L1–PD-1 is protecting the peripheral tissues from bystander or antigen-specific destruction mediated by activated effector T cells [86].

Engagement of PD-1 in T cells by PD-L1 during an immune response is bidirectional: on one hand, downregulated CD8 T cell functions induce “CD8 T cell exhaustion”, an anergic phenotype, and in the end apoptosis of tumor-specific T cells which express a high level of PD-1 [87,88] and on the other hand PD-L1 can deliver an anti-apoptotic signal in cancer cells that prevents apoptosis [30].

More recently, it has been shown that PD-L1 expression on DCs promotes the induction of adaptive Foxp3^+^CD4^+^ regulatory T cells (aTregs), and PD-L1 is a potent inducer of aTregs within the tumor microenvironment [89]. PD-1 blockade reversed the increased expression of PD-1 and PD-L1 on human melanoma antigen-specific CTL by Tregs, rescued INF-γ expression by melanoma antigen-specific CTL that were diminished by Tregs, and resulted in the downregulation of intracellular FoxP3 expression by Tregs [90].

**Figure 1:**
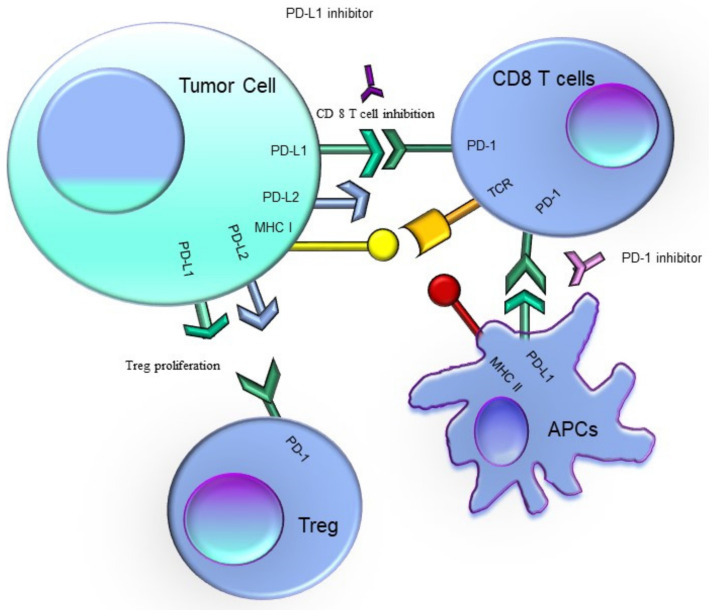
PD-1–PD-L1/2 pathway mechanism in cancer and under physiologic conditions.

Blockade of the interaction between PD-1 and PD-L1 potentiates immune responses in vitro [91] and mediates antitumor activity [87]. PD-L1, the main PD-1 ligand, is overexpressed in solid tumors and inhibits cytokine production and the cytolytic activity of PD-1+, tumor-infiltrating T cells [92,93]. Anti-PD-L1 antibodies block the interactions between PD-L1 and both PD-1 and CD80 (Figure 1) [31,32,94]. Based on these observations, PD-L1 inhibitors have been tested, and later approved in the treatment of different types of cancers.

In addition, elevated levels of soluble PD-L1 (sPD-L1) were produced and released by activated mature dendritic cells (mDC), and sPD-L1 has been identified in cancer patients and was associated with increased cancer-related death; tumor-derived or mDC-derived sPD-L1 was active, inducing apoptosis in T cells [95,96]. Higher levels of sPD-L1 were associated with larger or more advanced stage or grade tumors, and thus with an increased risk of death [95,96].

## 4. IL-10 Increases PD-L1 on Malignant Cells

Recent studies have suggested the existence of a correlation between the PD-1–PD-L1 pathway and the production of IL-10.

The triggering of PD-1 expressed on monocytes by PD-L1, expressed on various cell types, induced IL-10 production in monocytes, which in turn led to reversible CD4 T cell dysfunction in HIV-infected subjects [97]. Furthermore, autocrine IL-10 released by activated monocytes or resting monocyte-derived macrophages (MDM) increased PD-L1, but not PD-L2, on monocytes and MDM [18,97,98,99]. The basal levels of IL-10, produced by resting MDMs, were sufficient to suppress the expression of PD-L2, and IL-10 blockade enhanced only PD-L2 expression.

Moreover, a comparison of the kinetics of cytokine production [100] and PD-L1 expression [99] in hepatocellular carcinoma cell culture supernatant (TSN)-treated monocytes revealed that the accumulation of TNF-α and IL-10 preceded the upregulation of PD-L1, suggesting a novel immune-editing mechanism by which tumors increased the suppressor activity of activated monocytes by stimulating IL-10 and PD-L1 expression [99].

Monocytes, stimulated with breast cancer supernatant, showed increased expression of IL-10, IL-8, and chemokines CCL17 and CCL22, which are associated with an alternatively activated phenotype (aaMΦ); aaMΦ inhibits T cell proliferation [101] and their presence in breast carcinomas correlates with poor prognosis in patients [102].

Limiting IL-10 can dramatically enhance type 1 immune responses generated by protein and TLR-ligand-based vaccine formulations, improving vaccine design in humans [103]. In vitro blockade of either IL-10 or PD-L1 increased hepatitis C virus (HCV) specific T cell responses [104,105,106]. Brooks et al. demonstrated that in mice, IL-10 and PD-L1 suppress antiviral T cell activity via separate pathways and consequently, simultaneous blockade of IL-10 and PD-L1 dramatically increases T cell responses over that seen by neutralizing either molecule alone, and the combinatorial blockade of both IL-10 and PD-L1 rapidly eliminates persistent virus infection [107]. The blockade of the PD-L1–PD-1 and IL-10 axis in vitro in peripheral blood mononuclear cells (PBMC) from HIV-1-infected persons, and blockade of PD-1 in vivo in a SIV-infected macaque model have produced promising results [108,109].

To overcome the immunosuppressive effect of IL-10 on APC such as DC, Diaz-Valdes et al. [110] developed peptide inhibitors of IL-10, and the results suggest that IL-10 inhibiting peptides may have important applications to enhance anti-HCV immune responses by restoring the immunostimulatory capabilities of DC. Peptide inhibitors of IL-10 signaling, either directed to IL-10 or the IL-10R, have advantages such as lower manufacturing cost, higher activity per unit mass, greater stability for storage, better organ penetration, and the possibility of sequence modification to improve activity, half-life, and specificity, and they might be useful in cancer treatment [110].

## 5. PD-1/PD-L1 in AML

AML is mostly a disease of the elderly, and it is characterized by a clonal expansion of myeloblasts, which consecutively leads to bone marrow failure. From the discovery of anthracyclines and cytarabine in the 1970s, the 5-year OS slowly improved from 13% to 49% in young patients and from 8% to 13% in elderly patients [111]. PD-1/PD-L interactions play an important role in hematologic malignancies such as AML [83,112,113,114].

PD-1 was found to be abundantly expressed in leukemia patients and the frequency of PD-L1+ cells in AML was between 25% and 56% [115,116,117]. PD-L1 was significantly expressed in AML cells and was strongly enhanced after differentiation to dendritic-like leukemia cells (DLLC) [118]. A significant decrease in IL-12 production, increase in IL-10 production by DLLC, and an increased CD4^+^CD25^+^Foxp3^+^ T regulatory population led to the defective T cell immune response that was induced by PD-L1 upregulation in DLLC [118]. Blockade of PD-L1 expressed in DLLC results in increased T cell proliferation, Th1 cytokine production, specific cytotoxicity against AML blasts, and decreased Th2 cytokine production. PD-L1 downregulation was also proportional to the level of CD80. Some studies suggest that a higher PD-L1 expression is correlated with a worse prognosis [119] and with a higher rate of refractory/relapsed (R/R) disease [120].

Berthon et al. showed in a clinical trial with 79 AML patients that in 18% of cases, PD-L1 was expressed in more than 30% of the blasts [117]. No correlations between PD-L1 expression and AML subtype, age, molecular biology, or karyotype were found [117]. On the other hand, Zhang et al. suggested that a higher expression of PD-L1 is correlated with the M5 AML subtype [120], and Yang et al. suggested that a higher expression of PD-1 is associated with increased age [121]. PD-L2, less observed in AML patients (12.9%), was associated with the female gender when overexpressed [116]. Interestingly, a study on 197 AML patients showed in the subset analysis that PD-L1 expression is associated with the adverse group based on molecular biology and/or cytogenetics, and it is negatively correlated with TP53 [122]. The expression of PD-L1 increased when blast cells from patients with AML were exposed to the immune response or pathogens, and sometimes upon relapse. These findings suggest that PD-1/PD-L1 may be possible targets for immunotherapy via small molecules [112], while the low expression of PD-L2 makes it a less attractive target [123].

IFN-γ or TLR ligands induced PD-L1 expression, suggesting that various stimuli, either produced during the immune response against leukemia cells or released by infectious microorganisms, could protect leukemic cells from cytotoxic T cells by inducing PD-L1 expression [117]. PD-L1 cell surface expression was significantly upregulated (>20% PD-L1+ cells) by IFN-γ/TNF-α treatment in AML cells of 7 out of 10 newly diagnosed patients, whereas the expression of PD-L2 was only slightly induced. PD-L1-expressing AML cells displayed very low expression of CD80 and a variable expression of CD86, which was not influenced by IFN-γ/TNF-α treatment [19].

Another interesting function of PD-L1 is a selective co-stimulation of IL-10 secretion in both human and mouse T cells in the presence of anti-CD3 as a surrogate T cell receptor (TCR) signal [124]. PD-L1, expressed by either malignant cells or tumor-infiltrating DC, has been shown to promote the development, maintenance, and suppressive functions of Tregs in diverse hematologic malignancies such AML [114,125,126].

A study on a murine AML model showed that tumor progression is associated with high levels of Tregs and the over-expression of PD-1 on CD8 CTLs in the tumor. Thus, the interaction between PD-1 and PD-L1 suppresses T effector cells and the response towards the blast cells. [114]. PD-L1 inhibitors increased the proliferation and function of CTLs at tumor sites, decreased the tumor burden, and thus translated into a better OS. Treg depletion followed by PD-1/PD-L1 blockade showed superior efficacy for the eradication of established AML [114].

Studies have shown that the frequencies of marrow and blood Tregs are greater in patients with AML than in control patients [127,128,129].

In some studies, IL-10 levels were reported to be increased in AML patients [68,130], and some studies reported that patients with higher levels of IL-10 had more frequently attained remission [68,69]. The association of high levels of IL-10 with longer survival times can be explained by its capacity to inhibit granulocyte and granulocyte–macrophage colony-stimulating factor production, and, consequently, blast proliferation. IL-6, IL-1β, and TNF-α are cytokines that have been associated with lower survival in different diseases [130].

DNA demethylation is correlated with PD-1 overexpression and with a lower ORR and OS. Thus, other studies demonstrated that hypomethylating agents lead to the overexpression of PD-1, PD-L1, PD-L2, and CTLA4 [131,132].

Even though the association of venetoclax with hypomethylating agents improved the OS of elderly patients with AML, these results are not yet satisfying [133]. Thus, later studies developed the combination of venetoclax + azacitidine/decitabine + a PD-1 inhibitor as a first- or second-line treatment. The combination of venetoclax + azacitidine + pembrolizumab in the first-line setting in AML patients is currently under evaluation in the Blast-MRD-AML-2 study [134]. The combination of azacitidine + nivolumab showed efficacy and safety as a first-line therapy in elderly, hypomethylation-naïve patients [135]. Azacitidine + pembrolizumab was studied in R/R AML patients but also in newly diagnosed elderly patients. Out of the 37 R/R patients, 4 were in CR, 2 in complete remission with incomplete recovery (CRi), 1 in partial remission (PR), and 7 in stable disease (SD), for six cycles or more. In the other cohort of elderly patients, from the 22 patients included in the study, 8 patients were in CR/CRi, 2 in PR, and 4 had SD [136]. Azacitidine was also evaluated in combination with durvalumab or alone, in a first-line setting, in patients with high-risk myelodysplastic syndrome (HR-MDS) or AML, but no statistical difference between the two cohorts was observed [137]. Further results are also awaited from the Blast-MRD-AML-1 study, which evaluated high-dose chemotherapy + pembrolizumab in AML patients [138]. Decitabine was also evaluated with pembrolizumab in R/R AML patients. At the end of cycle 8, out of the 10 patients enrolled, 1 achieved CR, 4 SD, 4 progressed, and 1 was withdrawn from the study due to septic complications [139]. In a small clinical trial, 6 of the 13 patients with AML achieved CR/CRi/complete remission without platelet recovery (CRp) with the combination of azacitidine, nivolumab, and ipilimumab (a CTLA4 inhibitor) [140].

Interestingly, a meta-analysis comprising 19 studies showed that treatment with a PD-1 inhibitor is superior to a PD-L1 inhibitor, mostly because PD-1 inhibitors block both ligands compared to PD-L1 blockers, which allow tumor cells to escape via the PD-1–PD-L2 axis [141].

For leukemia, relapses after allogeneic hematopoietic stem cell transplantation, few therapeutic approaches are available. One of them is the addition of PD-1/PD-L1 inhibitors, which can enhance the response of the immune system, triggering a powerful graft versus leukemia reaction (GVL) [142]. The early addition of these drugs after transplant can lead to GVL but with a higher risk of graft versus host disease (GVHD) compared to their addition later, which lowers this risk [143].

Although the role of IL-10 in AML needs to be clarified, the addition of immunomodulatory agents that block the IL-10 and PD-1 signaling pathways may be an interesting approach for the treatment of AML, but only in selected patients, probably in combination with other agents. In the future, triple or quadruple combinations may be available. As mentioned previously, there are already several studies that have assessed the combination of PD-1 and CTLA4 inhibitors and hypomethylating agents, with a good response, but in small cohorts. It would be also interesting to assess the benefits of the addition of other compounds such as sterile alpha motif and HD-domain-containing protein 1 (SAMHD1) inhibitors, anti-leukocyte immunoglobulin-like receptor B (LILRB4) antibodies, or poly[ADP-ribose] polymerase 1 (PARP1) inhibitors [144]. The discovery of new drugs and new targetable mechanisms against AML will of course raise other questions, such as who will best respond to which treatment. These questions will be answered with the help of predictive and prognostic biomarkers, the identification of which is warranted.

## 6. Targeting the PD-1 Signaling Pathway in Cancer Restored Tumor-Specific T Cell Effector Functions

Studies have shown that blocking the PD-L1–PD-1 signaling pathway, in conjunction with other immune therapies, prevents tumor progression by enhancing antitumor CTL activity and killing the tumor cells, and have demonstrated safety [83,145]. The blockade of PD-1–PD-L1 interactions using clinical-grade human antibodies increases the proliferation and IFN-γ production of minor histocompatibility antigen (MiHA)-specific CD8 T cells when stimulated with PD-L1-expressing AML blast cells and DC, indicating that the PD-L1–PD-1 signaling pathway suppresses MiHA-specific CD8 T cell responses [19].

In the present, there are three PD-1 inhibitors (nivolumab, pembrolizumab, cemiplimab) and three PD-L1 inhibitors (avelumab, atezolizumab, durvalumab) approved by the FDA [146]. Additionally, there are several PD-1/PD-L1 inhibitors that are currently under investigation. KN035 (envafolimab) is the first subcutaneous PD-L1 inhibitor which has been investigated in phase I clinical trials, in the USA and China, as a single agent, for different solid cancers, and has shown a good safety profile and anti-neoplastic effect [147,148]. Additionally, envafolimab is currently being investigated in the ENVASARC trial with or without ipilimumab in solid tumors [149]. CK-301 (cosibelimab) is another PD-L1 experimental inhibitor currently being investigated in phase I clinical trials for different solid cancers. Cosibelimab showed a good safety profile and an ORR of 47% in a multicenter clinical trial [150]. Spartalizumab, a PD-1 inhibitor, was investigated for the treatment of melanoma and NSCLC [151]. Unfortunately, the COMBI-I trial showed that the addition of spartalizumab brought no benefit in the treatment of BRAF-V600-positive melanoma patients in association with dabrafenib and trametinib, compared with dabrafenib and trametinib. Other PD-1/PD-L1 inhibitors that are currently being investigated are BMS-936559, CS-1001, SHR-1316 (HTI-1088), CBT-502 (TQB-2450), and BGB-A333 [152].

The identification of predictive biomarkers will be important in the future selection of patients for therapeutic strategies targeting the PD-L1/PD-1/B7-1 axis.

PD-L1 expression in tumor cells might serve as a predictive or prognostic response to anti-PD-1 treatment, because patients with PD-L+ tumors are more likely to have tumor regression [93]. The combination of CLTA-4 and PD-1 blockade in the context of a whole-cell antitumor vaccine resulted in an increase in tumor-infiltrating T cells and a reduction in Tregs within the tumor [153].

## 7. Consequences of the Hypothesis and Discussion

Allogeneic hematopoietic stem cell transplantation provides a potent anti-leukemic effect, but novel strategies are needed for patients ineligible for this treatment. The manipulation of networks such as the co-inhibitory PD-1–PD-L1 pathway and suppressor IL-10–IL-10R pathway could be an attractive and novel immunotherapeutic intervention for AML patients ineligible for standard treatment with chemotherapy and hematopoietic stem cell transplantation. A crucial element to be taken into account is the selection of the “right” patients who would best respond to this combination, or, alternatively, this combination in association with other agents.

A greater knowledge of the effect of the combination of agents blocking PD-L1 and IL-10 molecules by themselves and with conventional therapies will benefit AML patients.

## Data Availability

Data sharing not applicable.
